# Diet Modification Alters Repeated Exposure Ethanol Tolerance Development

**DOI:** 10.17912/micropub.biology.002039

**Published:** 2026-03-19

**Authors:** Enrique Rodriguez Borrero, Laura Jimenez Marrero, Priscilla Gutierrez Ortiz, Adrian Rivera Alsina

**Affiliations:** 1 Biology, University of Puerto Rico at Cayey, Cayey, PR; 2 Natural Sciences, University of Puerto Rico at Cayey, Cayey, PR

## Abstract

Diet is a major environmental factor influencing alcohol‑related behaviors, yet its contribution to ethanol tolerance remains incompletely understood. Using a repeated‑exposure sedation assay in *Drosophila melanogaster* , we examined how high‑protein and high‑fat diets modulate the development of rapid and repeated tolerance. Flies on a standard diet exhibited both forms of tolerance, and dietary modification enhanced repeated tolerance acquisition. High‑fat feeding reduced survival, abolished rapid tolerance, and permitted only limited tolerance after repeated exposures. These findings demonstrate that diet differentially shapes ethanol sensitivity and tolerance, identifying high‑fat feeding as a potent disruptor of ethanol‑induced neuroadaptive responses in *Drosophila* .

**Figure 1. Survival outcomes and effects of diet modification on repeated ethanol tolerance development:**
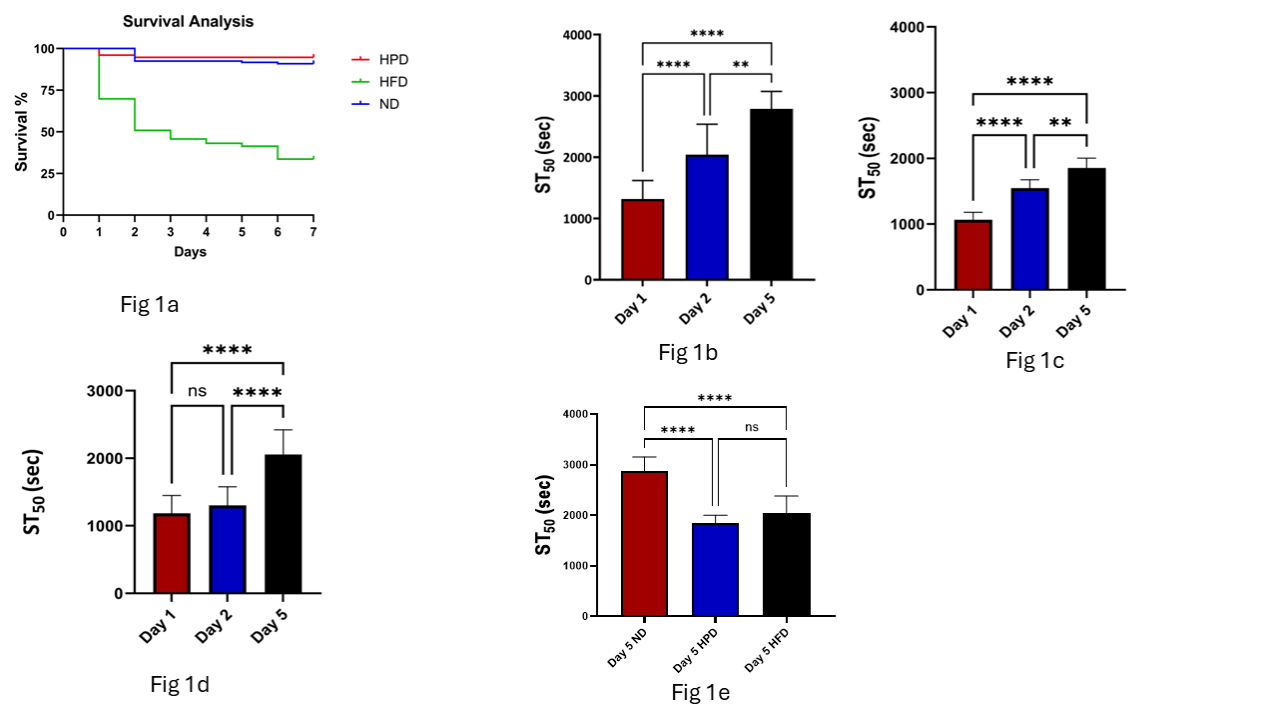
**(a)** Survival percentages were plotted for flies maintained on HPD (red), HFD (green), and Normal Diet (blue). Statistical analysis via log-rank test revealed significant differences between HFD and the other groups (p < 0.001), indicating a detrimental effect of high-fat intake on short-term survival. **(b)** SD₅₀ values (sec) measured on Day 1 (red), Day 2 (blue), and Day 5 (black) of control flies cultures flies. Bars represent mean ± SEM. Statistical analysis was performed using one-way ANOVA with Tukey’s post hoc test. SD₅₀ was significantly higher on Day 2 and Day 5 compared to Day 1 (****p < 0.0001), and Day 5 was significantly higher than Day 2 (**p < 0.01). **(c)** SD₅₀ values (sec) measured on Day 1, Day 2, and Day 5 of HPD cultured flies. Bars represent mean ± SEM. Statistical analysis was performed using one‑way ANOVA with Tukey’s post hoc test. SD₅₀ was significantly higher on Day 2 and Day 5 compared to Day 1 (****p < 0.0001), and Day 5 was significantly higher than Day 2 (**p < 0.01). **(d)** SD₅₀ values (sec) measured on Day 1 (red), Day 2 (blue), and Day 5 (black) of HFD cultures flies. Bars represent mean ± SEM. Statistical analysis was performed using one-way ANOVA with Tukey’s post hoc test. SD₅₀ was significantly higher on Day 5 compared to Day 1 and Day 2. No significant difference was observed between Day 1 and Day 2. **(e)** ST₅₀ values on Day 5 for ND, HPD, and HFD flies. HPD and HFD significantly reduced ST₅₀ relative to ND (****p < 0.0001), with no difference between HPD and HFD. Statistical analysis was performed using one-way ANOVA with Tukey’s post hoc test.

## Description

**Alcohol use disorder (AUD)** is a chronic brain condition characterized by impaired control over alcohol consumption despite adverse consequences. It encompasses diagnostic categories historically referred to as alcohol abuse, dependence, addiction, or alcoholism. AUD severity ranges from mild to severe, with persistent neuroadaptive changes caused by chronic alcohol misuse contributing to heightened relapse vulnerability (NIAAA, 2025).

Alcohol is among the most widely consumed psychoactive substances worldwide, in part due to its deep integration into social and cultural practices (Spanagel, 2009). Despite its ubiquity, the physiological and health effects of chronic ethanol consumption remain active areas of investigation. Repeated alcohol exposure leads to tolerance, defined as a reduced physiological or behavioral response to a given dose of ethanol (Elvig et al., 2021). Tolerance is a drug induced adaptation that can precede and overlap with the neurobiological processes underlying addiction. It may arise from alterations in ethanol pharmacokinetics-absorption, metabolism, or excretion from neuroadaptive changes at the cellular and molecular levels that reduce alcohol sensitivity. Tolerance is commonly categorized by timescale and mechanism: acute tolerance develops within a single exposure, rapid tolerance emerges after one exposure but persists beyond ethanol clearance, and chronic tolerance arises following repeated or prolonged exposure (Elvig et al., 2021; Koob & Bloom, 1988; Scaplen & Kaun, 2016). Chronic tolerance is intricately linked to physical dependence (Goldstein, 1983).

Studying AUD in mammalian models is challenging due to the complexity of their genomes, behaviors, and neural circuitry (Morales & Margolis, 2017). *Drosophila melanogaster* offers a powerful alternative: it shares approximately 75% of human disease associated genes (Chan & Bonini, 2000; White, 2016), possesses a compact but well-characterized nervous system, and provides extensive neurogenetic tools for precise circuit manipulation. Reward related neural circuits in flies are remarkably similar to those in mammals (Scaplen & Kaun, 2016), making *Drosophila* an excellent model for validating candidate genes and dissecting molecular pathways underlying AUD-related phenotypes (Engel et al., 2019).

Alcohol‑induced adaptations in *Drosophila* can persist for surprisingly extended periods. For example, flies that experience a single sedating exposure to ethanol vapor show enhanced tolerance: when re‑exposed to ethanol, they recover from intoxication more rapidly, and this effect can still be detected 1–2 weeks after the initial exposure (Cowmeadow et al., 2005). Flies also exhibit withdrawal like symptoms including ethanol-induced neuronal hyperexcitability, paralleling responses observed in humans (Bayard M et al., 2004; Ghezzi et al., 2014). Genome-wide association studies (GWAS) in *Drosophila* have identified numerous genes with mammalian orthologs linked to alcohol related behaviors (Lathen et al., 2020).

Beyond genetics, diet strongly influences alcohol related phenotypes. Dietary composition affects locomotor activity, feeding behavior, and lifespan (Ormerod et al., 2017). Epidemiological evidence suggests that individuals consuming high fat diets (HFDs) are more likely to escalate alcohol intake, promoting tolerance development (Coker et al., 2021). In *Drosophila* , HFDs enriched with saturated fats, sugars, and salts reduce lifespan, locomotor activity, and circadian rhythmicity (Murashov et al., 2021). Ethanol exposure itself disrupts circadian gene expression (De Nobrega & Lyons, 2016; Pohl et al., 2013), highlighting potential interactions between diet and ethanol in shaping tolerance mechanisms.

Experimental HFD formulations in flies commonly include coconut oil, lard, or palm oil (Woodcock et al., 2015, Gáliková & Klepsatel, 2018 Rivera et al., 2019; Murashov et al., 2021;). These diets activate intestinal stem cells in a microbiota-dependent manner (von Frieling et al., 2020) and reduce gut microbiota diversity in mammals, influencing cholinergic signaling (Chopra et al., 2022). Conversely, high protein diets (HPDs) enriched with yeast enhance ethanol resistance through serotonergic neuron activity, with effects emerging within two days and persisting for at least four days (Schmitt et al., 2020).

Together, these findings underscore diet as a critical environmental factor shaping alcohol tolerance. While mammalian outcomes vary due to neurotransmitter complexity (Coker et al., 2020; Sirohi et al., 2017), *Drosophila* provides a simplified yet genetically tractable system for dissecting diet-induced modulation of alcohol tolerance.

**In this study, we investigated how dietary modification influences AUD related phenotypes by exposing *Drosophila melanogaster* to high-protein and high-fat diets and assessing their effects on ethanol tolerance acquisition over repeated exposures.**

To evaluate the impact of dietary modifications on fly viability, we performed a Kaplan–Meier survival analysis. As shown in Fig. 1a, flies maintained on an HFD for seven days exhibited a marked reduction in life expectancy, indicating that HFD exposure poses a significant survival risk. In contrast, flies fed a control diet or HPD showed minimal changes in survival probability, suggesting that these conditions do not compromise viability.

We next applied our repeated alcohol exposure protocol to flies maintained on the control diet. As expected, these flies displayed typical ethanol tolerance dynamics. &nbsp;Repeated exposure tolerance emerged after five consecutive exposures, evidenced by an increased sedation threshold ST50 on day 5 (Fig. 1b). Rapid tolerance was also observed, reflected by the rise in ST50 between the first and second exposures, indicating a rapid adaptive response to ethanol. These findings suggest cumulative behavioral modulation or tolerance development over repeated exposures.

To determine whether dietary modifications influence ethanol tolerance development, we evaluated flies exposed to HPD and HFD. Flies on HPD (Fig. 1c) developed Repeated ethanol tolerance, as indicated by a higher ST50 on day 5 compared to day 1. Rapid tolerance was also evident, with an increase in ST50 between the first and second exposures. Strikingly, flies exposed to HFD (Fig. 1d) developed repeated but not rapid ethanol tolerance, suggesting that HFD impairs the physiological mechanisms required for ethanol adaptation. Repeated exposure increased ethanol sensitivity on HPD and HFD when compared to ND on the 5 ^th^ day (Fig. 1e). &nbsp;

Our results show that dietary composition significantly shapes ethanol‑related adaptations in *Drosophila melanogaster* . Flies on a standard diet developed both rapid and repeated tolerance, consistent with previous studies demonstrating robust ethanol‑induced plasticity in this model (Cowmeadow et al., 2005; Larnerd et al., 2023; Sandhu et al., 2015). High‑protein feeding similarly supported tolerance development, aligning with reports that protein‑rich diets enhance ethanol resistance through neuromodulatory pathways (Schmitt et al., 2020; Zhao et al., 2022). Despite this, HPD‑fed flies reached 50% sedation more rapidly than controls on day 5, suggesting increased accumulation of ethanol sensitivity.

In contrast, high‑fat feeding impaired ethanol adaptation. HFD‑fed flies failed to develop rapid tolerance and exhibited reduced survival, indicating that high‑fat intake disrupts metabolic or neurophysiological processes required for rapid ethanol‑induced plasticity. HFD is known to alter gut homeostasis, microbiota composition, and neuronal signaling (Jung et al., 2018; Murashov et al., 2021; Rivera et al., 2019; Sirohi et al., 2017; von Frieling et al., 2020), any of which may interfere with ethanol absorption, metabolism, or neuroadaptation. Although limited tolerance emerged after repeated exposures, the &nbsp;delayed tolerance formation suggests broad deficits in ethanol‑responsive pathways. Similar interactions between high‑fat diet, neural function, and alcohol‑related behaviors have been reported in humans (Coker et al., 2021).

Together, these findings identify diet as a potent modulator of ethanol sensitivity and tolerance. High‑fat diet emerges as a strong disruptor of ethanol‑induced neuroadaptation. The genetic and physiological tractability of *Drosophila* provides a powerful platform for future studies aimed at dissecting the molecular mechanisms linking metabolic state to alcohol responsiveness.

## Methods

**Materials and Methods**

**Fly Strains and Maintenance**

Wild-type *Drosophila melanogaster* Canton-S flies (Stock No. 64349) were obtained from the Bloomington Drosophila Stock Center (Indiana University, Bloomington, IN, USA). Flies were maintained at 25 °C, 50% relative humidity, under a 12 h:12 h light/dark cycle on standard Bloomington formulation diet (Nutri-Fly® BF, Cat. No. 66-112; Genesee Scientific, San Diego, CA, USA).

**Dietary Treatments**

Two- to five-day-old female flies were randomly assigned to one of three dietary treatments: control diet (CD), high-protein diet (HPD), or high-fat diet (HFD). Eleven female flies were placed in each Drosophila culture vial (VWR Cat. No. 75813-156), and each treatment was replicated eight times.

**High-Protein Diet (HPD)**

The HPD was prepared by supplementing the standard Nutri-Fly diet with 30% (w/w) autoclaved dry yeast (Red Star and Lesaffre Products, Milwaukee, WI), following Schmitt et al. (2020) with minor modifications.

**High-Fat Diet (HFD)**

The HFD was prepared by supplementing the standard Nutri-Fly diet with 30% (v/v) pure refined coconut oil (Thermo Scientific Cat. No. 365475000), following (Liao et al., 2021). To prevent flies from adhering to the oily surface, a piece of moist filter paper was attached to the wall of each vial, and a small piece of plastic mesh was placed inside. Food vials were maintained in a horizontal position throughout the experiment.

Flies were maintained on their respective diets for seven days prior to testing.

**Survival Analysis**

**Survival analysis was conducted using six vials per diet condition (ND, HPD, and HFD).** Mortality was recorded daily to assess diet-induced toxicity. *Day 0* was defined as the first full day following transfer to the experimental diet, allowing for acclimation and establishing the initial number of flies at risk. From this point forward, mortality counts were performed once per day. Cumulative survival was visualized using Kaplan–Meier survival curves to evaluate survival probability across time.

Survival curves were generated in GraphPad Prism version 10.0.0 (GraphPad Software, Boston, Massachusetts, USA; https://www.graphpad.com ) using the Survival module, with time (days) displayed on the x-axis and percent survival on the y-axis. Each fly was treated as an independent subject. Mortality events were encoded as *event = 1* , accompanied by the time of death (e.g., a fly observed dead on Day 7 contributed a survival time of 6 days, event = 1). Flies alive at the end of the study were right censored with *survival time = 7 days, event = 0* , as survival beyond day 7 was not evaluated.

Comparisons among diet treatments were made using differences in curve shape, decline rate, and final survival magnitude.. From a toxicological standpoint, these survival profiles reflect early signatures of diet-induced stress and potential toxicity in *Drosophila melanogaster* , providing functional insight into how nutrient balance modulates short-term viability.

**Alcohol Tolerance Assay**

The effects of diet variation on repeated exposure alcohol tolerance were assessed as described by (Sandhu et al., 2015), with minor modifications. Briefly, flies were exposed for 5 consecutive days to 0.5 mL of 50% ethanol) (Fisher Scientific Cat. No. A995-4) injected into cellulose acetate plugs (VWR Cat. No. 89168-886) using 1 mL surgical syringes fitted with 22-gauge needles (BD Syringe REF 309659; Fisher Scientific Cat. No. 14-829-10D). **12 vials** with **eleven flies** per vial were used on all diet treatments. The number of flies in each vial was recorded before treatment.

At time zero (t₀), ethanol saturated plugs were inserted into clear vials (VWR Cat. No. 75813-156) and pressed gently to ensure that the saturated portion was inside the vial but not exposed. Assays were performed in an incubator (VWR Shel Lab model no. HNR32P8-8) equipped with four IP cameras connected to a network video recorder (NVR) for continuous monitoring.

Sedation was assessed at 2 min intervals (t₂, t₄, t₆, etc.) until all flies were sedated. At each time point, vials were gently tapped to knock flies to the bottom, and sedated individuals were counted. Flies were considered sedated if they (a) were immobile and lying on their backs for subsequent time points, or (b) displayed rapid wing movement or ethanol-induced seizures.

When ≥80% of flies were sedated, ethanol- or water-treated plugs were replaced with fresh ones to allow recovery for 2–3 h. After recovery, flies were transferred back to food vials and maintained under standard incubation conditions. Treatments were repeated for five consecutive days.

On days 1, 2, and 5, ethanol‑induced sedation was monitored over time. The time at which 50% of flies became sedated was defined as the sedation time 50 (ST₅₀). ST₅₀ values were calculated for each run, with all vials from a given treatment group included in the analysis. Ethanol sensitivity was quantified as the difference in ST₅₀ between treatment groups during the first ethanol exposure. Tolerance was defined as the change in ST₅₀ within a treatment group following repeated ethanol exposures, specifically after the second and fifth exposures in this study.. Statistical analysis was performed in GraphPad Prism version 10.0.0 (GraphPad Software, Boston, Massachusetts, USA; https://www.graphpad.com ).

&nbsp;

&nbsp;

&nbsp;

## Reagents

Fly strain used:

**Table:** 

Strain	Species	Resource Reference ID
Canton-S	*Drosophila melanogaster*	BDSC_64349

&nbsp;

Reagents&nbsp;&nbsp;&nbsp;&nbsp;&nbsp;&nbsp;&nbsp;&nbsp;&nbsp;&nbsp;&nbsp;&nbsp;&nbsp;&nbsp;&nbsp;&nbsp;&nbsp;&nbsp;&nbsp;&nbsp;&nbsp;&nbsp;&nbsp;&nbsp;&nbsp;&nbsp;&nbsp;&nbsp;&nbsp;&nbsp;&nbsp;&nbsp;&nbsp;&nbsp;&nbsp;&nbsp;&nbsp;&nbsp;&nbsp;&nbsp;&nbsp;&nbsp;&nbsp;&nbsp;&nbsp;&nbsp;&nbsp;&nbsp;&nbsp;&nbsp;&nbsp;&nbsp;&nbsp;&nbsp;&nbsp;&nbsp;&nbsp;&nbsp;&nbsp;&nbsp;&nbsp;&nbsp;&nbsp;&nbsp;&nbsp;&nbsp;&nbsp;&nbsp;&nbsp;&nbsp;&nbsp;&nbsp;&nbsp;&nbsp;&nbsp;&nbsp;&nbsp;&nbsp;&nbsp;&nbsp;&nbsp;&nbsp;&nbsp;&nbsp;&nbsp;&nbsp;&nbsp;&nbsp;&nbsp;&nbsp;&nbsp;&nbsp;&nbsp;&nbsp;&nbsp;&nbsp;&nbsp;&nbsp;&nbsp;&nbsp;&nbsp;&nbsp;&nbsp;&nbsp;&nbsp;&nbsp;&nbsp;&nbsp;&nbsp; Source

**Table:** 

Shel Lab Incubator Model #HNR32P8-8	VWR
*Drosophila* Diet, Bloomington Formulation	Genesee Scientific Cat No.66-112
*Drosophila* culture tubes PS 25x95mm	VWR Cat No.75813-162
*Drosophila* experimental clear vial PP 28.5x95mm	VWR Cat No. 75813-156
Droso Plugs Wide Vials	Genesee Scientific Cat No. 59-201
*Drosophila* clear vials Cellulose Acetate	VWR Cat No. 89168-886
Coconut Oil, Pure, Refined	Thermo Scientific Cat No.365475000
Propionic Acid	Fisher Scientific Cat No. 79-09-4
Alcohol Reagent HPLC Grade	Fisher Scientific A995-4
BD 1ml TB Syringe with BD PresicionGlide Needle 22G 22G x 1(0.7mm x 25mm)	BD Syringe REF 309659
Active Dry Yeast	Red Star and Lesaffre Products, WI

&nbsp;

&nbsp;
